# Cardiovascular Risk Factors Involved in Hemorrhagic Transformation After Intravenous Thrombolytic Therapy in Patients with Acute Ischemic Stroke

**DOI:** 10.3390/ijms262010186

**Published:** 2025-10-20

**Authors:** Ileana Neacă, Cristina Elena Negroiu, Iulia Tudorașcu, Raluca Dănoiu, Sânziana Godeanu, Suzana Dănoiu, Despina Manuela Toader

**Affiliations:** 1 Doctoral School, University of Medicine and Pharmacy of Craiova, 200349 Craiova, Romania; sanzianagodeanu@yahoo.com; 2 Sanocare Medical Center Craiova, 200061 Craiova, Romania; danoiuraluca@yahoo.ro; 3 Department of Pathophysiology, University of Medicine and Pharmacy of Craiova, 200349 Craiova, Romania; irtudorascu@gmail.com (I.T.); suzanadanoiu@yahoo.com (S.D.); 4 Clinical Emergency County Hospital of Craiova, 200642 Craiova, Romania; despinamtoader@yahoo.com

**Keywords:** acute ischemic stroke, thrombolytic therapy, hemorrhagic transformation, risk factors

## Abstract

In line with AHA/ASA guidance, intravenous alteplase has served as the standard first-line reperfusion treatment in acute ischemic stroke (AIS). Hemorrhagic transformation (HT) is a common spontaneous complication after thrombolytic therapy for AIS with increased mortality. Restoration of flow in an occluded artery can precipitate blood–brain barrier breakdown and heighten the risk of HT. However, the pathogenesis of HT is multifactorial, and identifying patients at high risk after recanalization therapy (RT) has a defining role in ensuring optimal treatment. At the same time, it is still under debate how these patients can best be identified based on clinical and biological characteristics. Preventing HT will become increasingly essential. In this review, our primary objective was to identify research focused on the cardiovascular risk factors predicting HT after AIS treated with thrombolytics, as this may help develop targeted treatment strategies and diminish the risk of HT.

## 1. Introduction

Acute ischemic stroke (AIS) represents the majority of strokes, at an estimated 60–80% [1,2]. Reperfusion therapy (RT) represents the mainstay of AIS treatments [3,4], and may be achieved pharmacologically with intravenous recombinant tissue-type plasminogen activator (rtPA; alteplase) [5] or, since 2015, via endovascular therapy (EVT) for anterior circulation large-vessel occlusion (LVO) [6]. Current guidance recommends administering IV rt-PA within 4.5 h of symptom onset [3,5,7,8], and achieving a door-to-needle time within 60 min [9]. Through reperfusion treatments, the functional outcome of patients is enhanced, and the 3-month neurological disability is reduced [10]. Even though the results are striking, in some situations, restoration of flow in an occluded vessel can facilitate BBB destabilization, which contributes to reperfusion injury and elevates the risk of hemorrhagic transformation (HT) [11,12,13,14]. Consequently, a notable proportion of patients experience unfavorable outcomes despite rt-PA treatment because of ensuing complications, the most important being symptomatic intracerebral hemorrhage (sICH) [15]. The rise in morbidity and mortality linked to the occurrence of hemorrhagic complications makes its prevention a highly relevant field of research [16].

Following AIS, HT is diagnosed when infarcted areas appear hemorrhagic on radiologic studies [17]. Despite the easily accessible diagnostic imaging, the ability to identify people at high risk of developing HT remains a real challenge. Risk factors for HT after intravenous thrombolysis form a broad spectrum, and although the extent to which they contribute remains unclear [17].

The aim of this review is to provide an up-to-date, integrated synthesis of HT after reperfusion therapy in AIS, beginning with imaging classifications and pathophysiological mechanisms and progressing to the most relevant cardiovascular risk factors, both modifiable and non-modifiable. The novelty of this work lies in linking mechanistic evidence with recent clinical data on blood pressure dynamics, hyperglycemia, and lipid profile/statin use, to derive practical implications and strategies for reducing hemorrhagic risk after rt-PA.

## 2. Methods

We conducted a narrative review of the literature on HT after reperfusion therapy for AIS, with a focus on cardiovascular risk factors and BBB dysfunction. A comprehensive search of PubMed/MEDLINE, Embase, Web of Science, and the Cochrane Library was performed for studies published from 1998 (modern thrombolysis era) through January 2025. Search strings combined controlled vocabulary and free-text terms for AIS and reperfusion (e.g., “acute ischemic stroke,” “intravenous thrombolysis,” “alteplase”), hemorrhagic outcomes (“hemorrhagic transformation,” “parenchymal hemorrhage,” “intracranial hemorrhage,” “hemorrhagic infarction,” “sICH”), cardiovascular exposures (“blood pressure,” “BP variability,” “pulse pressure,” “autoregulation,” “hyperglycemia,” “lipid,” “diabetes”), and mechanisms (“blood–brain barrier,” “HIF,” “matrix metalloproteinase). Reference lists of key reviews and guidelines (AHA/ASA, ESO/ESMINT) were hand-searched to identify additional studies.

We prioritized evidence from recent clinical guidelines, systematic reviews/meta-analyses, randomized or quasi-randomized trials, and large prospective cohorts; well-designed observational studies were included where trial data were unavailable or to address uncommon outcomes (e.g., PH2). Inclusion criteria were: (i) adult humans with AIS treated by IV thrombolysis (alteplase) with or without EVT; (ii) reporting HT outcomes (any ICH; HI/PH; PH2; or sICH defined by NINDS/ECASS/Heidelberg) or mechanistic endpoints relevant to BBB/HIF/autoregulation. Exclusion criteria were: pediatric populations; primary ICH/SAH without ischemia; case reports/series < 10 patients; conference abstracts without full text; non-English publications; and purely animal studies unless directly supporting human BBB/HIF mechanisms.

Two reviewers independently screened titles/abstracts and full texts and extracted data on study design, population, reperfusion modality/agent, definitions of HT/sICH, BP metrics (baseline and post-tPA trajectories, variability indices), glycemic and lipid parameters (including statin exposure), and clinical outcomes (early neurological deterioration, mortality, and 90-day mRS). Given heterogeneity in designs, definitions, and exposure metrics, we performed qualitative synthesis without meta-analysis, highlighting convergent findings and areas of inconsistency relevant to bedside BP, glucose, and lipid management after reperfusion.

## 3. Incidence and Classification

Given that HT exhibits distinctive features on cross-sectional imaging and considering the current availability and speed of these techniques, radiologic diagnosis of hemorrhage is generally straightforward [17]. According to the European Cooperative Acute Stroke Study (ECASS), HT on imaging is generally classified into hemorrhagic infarction (HI) and parenchymal hemorrhage (PH) with or without mass effect [18,19]. Large PH and sICH have excess mortality [20].

In the study by Zhang X. et al., post-thrombolysis hemorrhage occurred in 38.84% of patients, with intracranial hemorrhage in 10.97%; these rates align with prior reports [8]. Paciaroni, M. et al., included a broad consecutive patient series with AIS, and the incidence of HI was 9%, and PH was 3% [21]. The current proportion of HT after AIS ranges from 8.5 to 30%, in which 2.1–9.4% are symptomatic HT [22,23,24,25]. Some studies revealed that treatment with rtPA is linked to a 6–8% risk of sICH [26,27,28].

**Figure 1:**
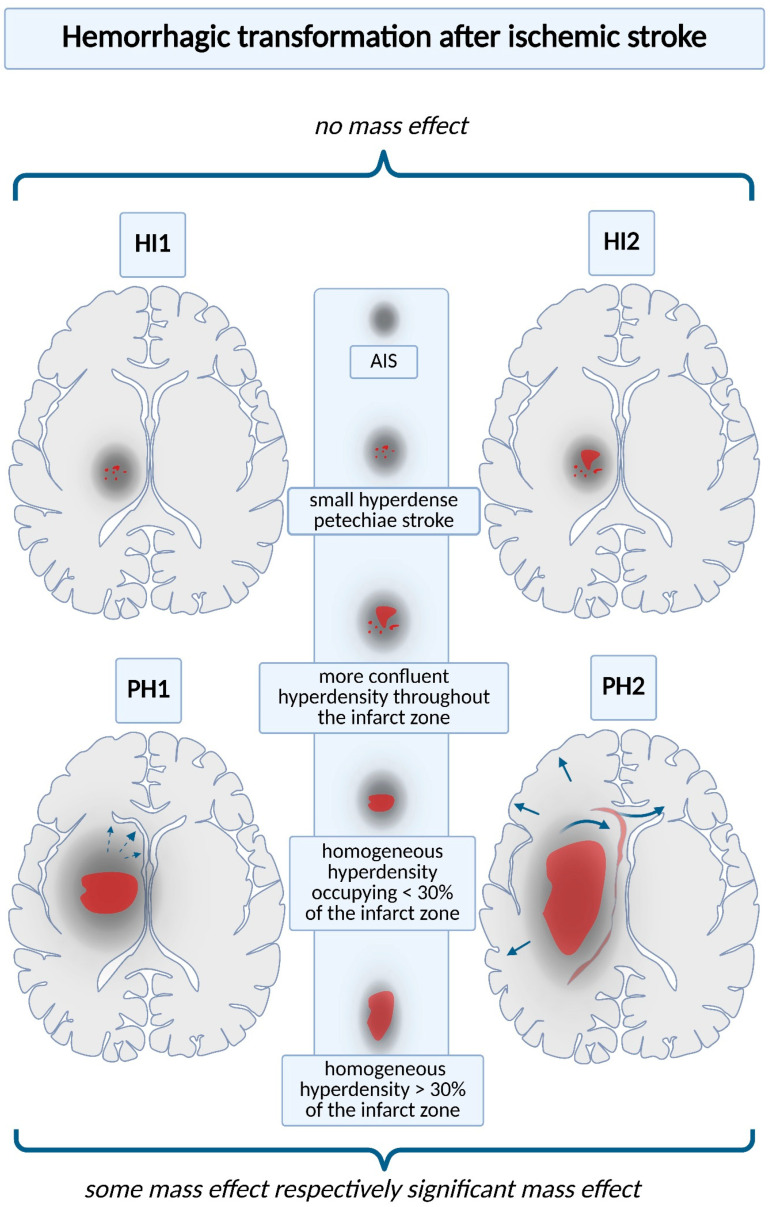
Hemorrhagic transformation after ischemic stroke. Each type of hemorrhagic complication is associated with an increased risk of subsequent complications; thus, accurate imaging definitions are necessary. On CT scans, they are normally classified into four categories: hemorrhagic infarction types 1 and 2 (HI1 and HI2); parenchymal hemorrhage types 1 and 2 (PH1 and PH2). HI1 presents as scattered, punctate hyperdense petechiae, whereas HI2 shows a more confluent hyperdensity extending across the infarcted territory. PH1 consists of a homogeneous hematoma involving < 30% of the infarcted area, typically accompanied by mild mass effect (documented by interrupted arrow). PH2 denotes a uniform hematoma occupying > 30% of the infarcted territory, typically associated with marked mass effect (documented by continuous arrows and exemplified by the reduction in cerebral gyri, compression of the ventricular system, and herniation phenomena).

HT includes a broad spectrum of severity, each form being associated with subsequent complications ranging from small petechial hemorrhagic infarcts (HIs) to PH (Figure 1) [10]. On computed tomography, HI presents as an irregular hyperdense area within a portion of the ischemic lesion. PH appears as a more homogeneous, hyperdense hematoma with associated mass effect. Each HT category comprises two subtypes: HI is classified as HI1 and HI2, while PH is divided into PH1 and PH2. HI1 manifests as small, speckled hyperdense spots (petechial hemorrhage); HI2 denotes a more extensive and confluent hyperdensity extending through the infarcted tissue. HI1 and HI2 have no mass effect. PH1 presents as a homogeneous hyperdense hematoma involving < 30% of the infarcted territory, accompanied by mild mass effect. PH2 presents as a homogeneous hyperdense hematoma involving > 30% of the infarcted territory, with pronounced mass effect [29,30,31]. sICH and asymptomatic ICH after AIS have been differentiated, but the prognostic significance of asymptomatic ICH remains largely unknown [10].

The definitions of sIH used are widely variable and take into account the radiological classification of hemorrhage and the degree of neurological deterioration [12]. According to the National Institute of Neurological Disorders and Stroke (NINDS) Stroke Intravenous Thrombolysis Study criteria, sICH denotes clinical deterioration occurring within 36 h after reperfusion therapy, accompanied by a CT-confirmed intracranial bleed [32].

Of note, the patients who are at high predicted risk for sICH are also likely to have very poor outcomes without thrombolytic therapy [28].

An additional class includes intracerebral hemorrhage not confined to the infarcted region, as well as extra-parenchymal intracranial bleeding, specifically subarachnoid hemorrhage and subdural hematoma [30]. There is ongoing debate regarding the prognostic implications of the various HT patterns after thrombolysis. PH2 is linked to clinical deterioration. HI often reflects reperfusion of marginally ischemic tissue and is commonly clinically silent [31,33,34]. PH could be linked to alteplase-related biological actions and to other disease processes that contribute to worsening clinical status [33].

## 4. Ischemic Stroke and Hemorrhagic Transformation: Cellular Pathology

**Figure 2:**
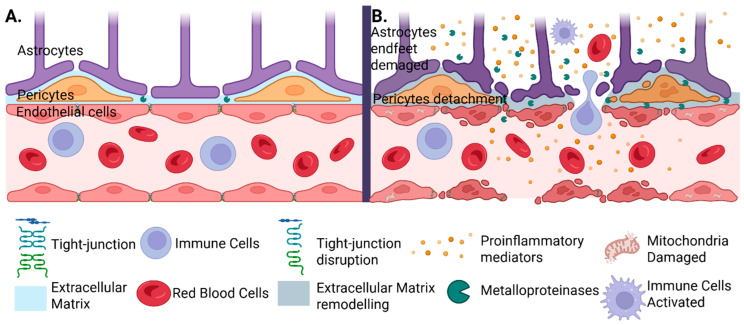
Structural and functional alterations of the blood–brain barrier (BBB) in cerebral ischemia. (**A**) Under physiological conditions, the BBB is maintained by the coordinated interaction between cerebral endothelial cells, pericytes, and astrocytic endfeet, supported by intact tight junctions and a stable extracellular matrix. (**B**) Cerebral ischemia or hemorrhagic transformation following thrombolysis may disrupt this balance. The release of matrix metalloproteinases (MMPs) affects not only the pericytes, causing them to detach, but also produces astrocytic endfeet damage, tight junction degradation, and extracellular matrix remodeling. This is followed by the infiltration with activated immune cells, pro-inflammatory mediators, and red blood cells, which promote extravasation. Mitochondrial damage further contributes to cellular dysfunction and the progression of injury.

Stroke occurs when nerve cells within a focal brain region (the infarct core) are deprived of nutrients and oxygen for at least 4 min [35]. This deficit leads to decreased ATP concentration, cellular acidosis, increased intracellular calcium concentration, the appearance of free radicals, inflammatory cytokines, glial activation, and destruction of the blood–brain barrier (BBB) with the presence of leukocyte infiltrate, finally leading to nerve cell death [36,37] (Figure 2). The ischemic zone includes two concepts, namely the infarct core and the penumbra zone, the differentiation of the two presenting an important clinical significance. The penumbra zone is the one that, even if it is prone to temporal changes, contains cells with disrupted electrical activity and potassium dysregulation that can be saved under intervention [35]. Clinical trials have shown that patients with smaller infarct cores and larger penumbral regions respond more favorably to treatment than those with extensive infarct sizes [32].

Neuronal loss after ischemia is believed to involve both apoptotic and necrotic mechanisms as core drivers. Reducing oxygen and nutrient supply to the brain triggers anaerobic glycolysis, where pyruvate is converted to lactate with the release of protons (H+), leading to a decrease in cellular pH. Simultaneously, increased pCO2 exacerbates the situation by promoting hydration to form carbonic acid, thereby enhancing acidosis [38].

A key mediator of cellular adaptation to low oxygen is HIF-1, which coordinates the expression of a broad gene network involved in both protective and deleterious processes [39,40]. HIF-1α increases VEGF-A expression, which enhances endothelial permeability, downregulates tight-junction proteins, and promotes plasma extravasation [41]. It also stimulates MMP-2 and MMP-9, which cleave tight-junction proteins and degrade the basement membrane, thereby destabilizing the blood–brain barrier [42]. HIF-1α interacts with pro-inflammatory pathways and reactive oxygen species, amplifying cascades that injure the endothelium and increase blood–brain barrier permeability [43]. Although acutely harmful, HIF-1α may promote regenerative processes such as angiogenesis and neurogenesis during prolonged or moderate hypoxia, underscoring its context-dependent role [44].

Simultaneously, reduced ATP levels lead to impairment of ATP-dependent ion pumps within the infarct core [45]. A key research focus has been understanding the mechanisms behind this ATP depletion and the resulting pump dysfunction. In response to the low oxygen levels, cells experience a sustained increase in the concentration of free cytosolic Ca^2+^ and Na^+^, driven by the excessive neuronal glutamate release [46]. An increase in Na^+^ levels harms neuronal cells, but growing evidence highlights Ca^2+^ as a significant contributor to post-ischemic cellular death [35]. The rise in Ca^2+^ levels triggers the activation of calpains, caspases, free radicals, nitric oxide, and enzymes involved in arachidonic acid metabolism. This leads to protein cleavage and plasma membrane rupture while simultaneously activating the pro-apoptotic protein BID (BH3-interacting domain death agonist) [47].

Concurrent rises in cytosolic Ca^2+^ and reactive oxygen species trigger opening of the mitochondrial permeability transition pore (MPTP) in the inner mitochondrial membrane. Pore activation collapses cellular energetics and impairs mitochondrial function, leading to mitochondrial swelling, cytochrome-c release, and engagement of both apoptotic and necrotic death programs [35].

Compromised cells in both the core and penumbral regions emit signals for initiating post-ischemic inflammation [37]. This inflammation significantly influences the degree of neuronal damage resulting from ischemia and reperfusion. It triggers the activation of platelets, complement factors, and endothelial cells, initiating the coagulation cascade [35].

Within minutes of an ischemic insult, *p*-selectin and fibrin translocate to the surfaces of platelets and endothelial cells, initiating pro-inflammatory signaling cascades. This triggers the release of adhesion molecules (ICAM-1, VCAM-1), pro- and anti-inflammatory cytokines (e.g., IL-1β, IL-6, IL-10, IL-17, IL-22, TNF), and the induction of MMPs (MMP-2, -8, -9) and prostaglandins [48]. Additionally, chemokines such as CCL2, CXCL8, and CXCL10 orchestrate leukocyte recruitment [49]. All these mechanisms lead to alterations in the integrity of the BBB [28,50,51]. Compromise of the BBB together with impaired cerebrovascular autoregulation increases susceptibility to hemorrhagic extravasation when ischemic tissue is reperfused after thrombolytic therapy [52]. The extent of anatomical and physiological injury is strongly contingent on the duration of ischemia [53]. Beyond fibrinolysis, alteplase can facilitate HT by engaging immunologic mechanisms [54].

## 5. Clinical Outcome

The clinical outcome is not the same for all types of HT; many studies have shown that HT does not influence the patient’s prognosis. However, this has only been shown in the case of mild to moderate HT, where such hemorrhage may indicate a successful thrombolytic treatment and vascular recanalization [55]. PH2 is a strong predictor of neurological worsening and death [56,57]. The risk of early neurological deterioration and of 3-month mortality was severely increased after PH2 [58].

## 6. Cardiovascular Risk Factors

Cardiovascular risk factors usually present in AIS patients are age, arterial hypertension, diabetes mellitus, dyslipidemia, smoking, male sex, and obesity (Table 1).

### 6.1. Age

With advancing age, the walls of blood vessels become thinner and stiffer, due to the loss of elastin and the accumulation of collagen [77,78]. Arteriosclerosis and microangiopathy develop, making vessels more susceptible to rupture in the context of rapid recanalization following thrombolysis [79].

Zhang, J. et al. identified that age and blood glucose level were involved in HI [57]. Advancing age is linked to persistent elevation of inflammatory cytokines and alterations in immune response [13]. Such inflammatory markers help clarify how clinical factors drive HT risk, and they may offer novel therapeutic targets [13]. The ECASS 2 trial showed PHs were more frequent among elderly patients on aspirin at stroke onset after IV tPA treatment [80].

Multiple studies have demonstrated that older age independently increases the likelihood of HT after thrombolysis with alteplase. For example, the systematic prediction model from Hua et al. reported an odds ratio (OR) of approximately 1.5 for each age increment [59], while the study by Hao et al. identified age as a key predictor for parenchymal hemorrhage after IVT (OR = 1.038 per year, *p* < 0.001) [56]. Moreover, meta-analyses conducted by Sun et al. [81] and the mechanistic reviews by Hong et al. [17] confirm the strong link between age and susceptibility to BBB dysfunction and fragile tissue reperfusion.

**Figure 3:**
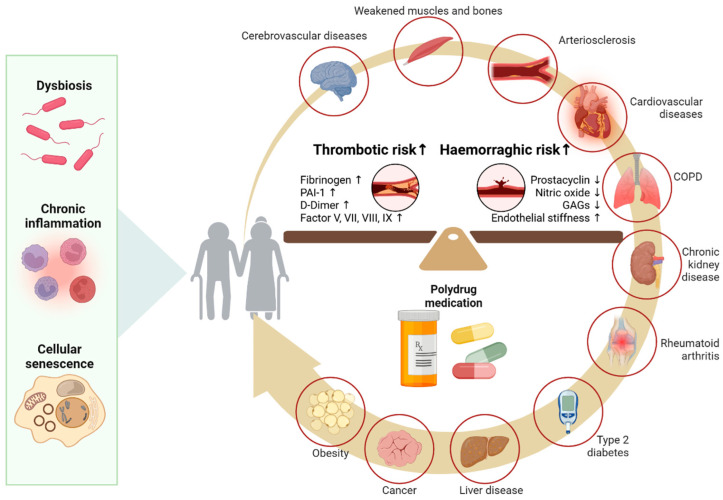
Age and blood-barrier dysfunction. As the body ages, molecular and cellular changes such as gut dysbiosis, chronic inflammation, and cellular senescence contribute to endothelial dysfunction and imbalances in the coagulation and fibrinolytic systems. These mechanisms are further aggravated by common comorbidities in the elderly (e.g., cardiovascular diseases, kidney disease, diabetes) and polypharmacy. PAI-1 (Plasminogen Activator Inhibitor-1) GAGs (Glycosaminoglycans).

The presence of multiple comorbidities in the elderly may also contribute to the higher incidence of sICH [10]. Aging also interferes with the coagulation cascade, increasing the risk of both bleeding and thrombotic risks due to endothelial dysfunction and changes in coagulation and fibrinolytic proteins. Hemorrhagic risk is favored by increased endothelial wall stiffness and decreased production of prostacyclin, nitric oxide, and glucosaminoglycans. The elevated levels of fibrinogen, coagulation factors V, VII, VIII, and IX, D-dimer levels, increased plasminogen activator inhibitor-1 (PAI-1), and an enhanced platelet response to ADP and collagen are, on the other hand, responsible for the increased thrombotic risk [82] (Figure 3). Under these conditions, alteplase acts on plasminogen and increases the risk of hemorrhage [61].

Gut microbiota has also emerged as a relevant factor in cerebrovascular disease. Age-related dysbiosis—characterized by a decrease in beneficial microbial diversity and an increase in pro-inflammatory species—is linked to systemic inflammation, endothelial impairment, immune dysregulation, and overall increased ischemic stroke risk [83]. Dysbiosis may also influence the integrity of the BBB through pro-inflammatory cytokines and microbial metabolites such as trimethylamine-N-oxide, thereby increasing susceptibility to both ischemic injury and HT post-thrombolysis [84].

### 6.2. Arterial Hypertension

A cerebral blood flow (CBF) around 50–60 mL per 100 g of tissue per minute is considered optimal in normal conditions [85]. In the penumbra, the CBF is reduced by 50%, leading to the cessation of synaptic transmission and a corresponding 50% decrease in cerebral energy consumption [86]. At this stage, blood flow can only be adjusted by ensuring adequate systemic perfusion [86]. Cerebral autoregulation is thus overwhelmed, resulting in the loss of the brain’s capacity to preserve adequate cerebral perfusion [87]. Consequently, a rapid reduction in systolic blood pressure during AIS may compromise perfusion to the ischemic area, potentially enlarging the infarct [87].

Blood pressure (BP) and its repercussions on the risk of developing hemorrhage before thrombolytic treatment are under debate. Many studies identified hypertension as an independent risk factor for post-thrombolysis and negatively correlated with stroke prognosis [17,56,88,89,90]. Over 60% of patients with acute stroke showed elevated BP levels [91]. The cause is multifactorial, with cumulative effects of neuroendocrine activation, impaired central autonomic control, and disrupted baroreceptor reflexes [92].

The increased BP has a primary mechanical impact at the vessel level within the brain, is thought to exacerbate inflammation, induce vascular remodeling, and alter collateral flow, endothelial function, and cerebral autoregulation [62].

The elevated BP may result from a constellation of factors:

- Central autonomic dysregulation.
- Acute sympathoadrenal (stress) surge.
- Pre-existing or uncontrolled hypertension.
- Neuroendocrine activation with downstream inflammatory cascades.
- Raised intracranial pressure [13].

Some studies found that elevated BP was associated with increased hemorrhage risk before administration when treated with TT [93]. The BP is an important prognostic determinant in hyperacute ischemic stroke patients receiving IV tPA. Peri-tPA systolic hypertension impacts the occurrence of HT and the overall clinical outcome [93]. The risk of hemorrhage after IV thrombolysis increased with higher systolic BP recorded before treatment [94,95].

Several studies suggest that the relevant predictor of PH is 24 h post-tPA systolic BP rather than pre-treatment SBP [96,97]. In Sun et al., pre-thrombolysis SBP was not an independent predictor, whereas higher systolic levels measured two hours after treatment were associated with increased risk of HT [98]. The studies supporting these observations are listed in Table 2.

The study by LEI, Y.-S et al. [99], retrospectively examined 220 consecutive patients with cerebral microbleeds treated with intravenous rt-PA within the first 4.5 h after the onset of AIS. Hypertension emerged as an independent risk factor for post-thrombolysis [99].

The guidelines recommend maintenance of BP below 185/110 mmHg to mitigate the risk of HT in alteplase-treated AIS [3]. Across studies, each 10-mmHg increment in systolic BP within the 140–180 mmHg range was associated with a higher risk of HT [100]. These findings were corroborated during the first 24 h after IV tPA in the SITS-ISTR registry [100]. Elevated SBP (2–24 h post–IV tPA), treated as a categorical variable, was linearly related to sICH risk [100]. Lower blood pressure in the first 24 h is associated with more favorable outcomes and a reduced incidence of sICH [101].

SBP greater than 185 mmHg and DBP greater than 110 mmHg prior to the bolus independently increased the likelihood of sICH [102]. The EPITHET study confirmed a strong BP–PH relationship in the post-rtPA period [97].

#### 6.2.1. The Variability in Systolic Blood Pressure and Pulse Pressure

Variability in BP following intravenous tPA is clinically salient and has been independently linked to lesion growth, early clinical evolution, and 90-day outcomes [96,103,104]. The SITS investigators confirmed these findings and found a strong association between BP variability defined by successive variation within the first 24 h and the occurrence of sICH [105]. BP fluctuated markedly in the hyperacute phase of AIS, showing a U-shaped time course over the first day—higher at presentation, declining over several hours, and rising again thereafter [106]. Baseline SBP/DBP and fluctuations in BP during the first 24 h post-injection showed associations with larger ischemic lesions and more severe symptoms [97]. BP variability is independently associated with sICH [107]. The variability in the first 6 h [108] in patients exhibiting high SP increases the risk of HT following alteplase administration [89]. A wider systolic–diastolic gap during the acute phase of AIS was linked to reduced 3-month survival [109] and more severe neurological deficits within the first 7 days [110].

Evidence suggests that arterial stiffness is correlated with sICH after IVT [111]. Recent literature emphasizes that BP variability, not just absolute levels, significantly influences HT and outcomes post-thrombolysis. For instance, Liu et al. reported that SBP extremes in the first 24 h post-tPA are independently related to HT (OR ≈ 1.042) [112]. Sandu et al. further showed a higher risk of HT up to 73% in patients with SBP [113], highlighting the prognostic and therapeutic importance of targeting stable BP profiles.

Mechanisms

Changes in blood pressure damage cerebral autoregulation, a physiological mechanism that maintains blood flow within the brain tissue. The process is regulated by cerebrovascular resistance, regardless of changes in the body’s BP. Cerebral autoregulation is impaired within ischemic stroke tissue, rendering regional cerebral blood flow pressure-passive. Consequently, systemic arterial pressure directly influences local perfusion. The ischemic penumbra—viable but underperfused tissue—thus becomes vulnerable to systemic BP changes: reductions in blood pressure can precipitate critical hypoperfusion, whereas elevations may transiently augment perfusion but carry risks of hyperperfusion injury, edema, or HT [114].

Patients with AIS typically have decreased arterial compliance. An increase in pulse pressure (PP) augments transmural stress on weakened cerebral arteries, predisposing to hemorrhage [115]. Higher PP in AIS is associated with a poor prognosis, particularly in the long term [102,115,116,117].

However, the recent literature emphasizes that it is uncertain whether BP variability is a causal factor in HT and early neurological deterioration, or rather a marker of disease severity and impaired cerebral autoregulation. Both short-term and long-term fluctuations in BP, which may arise from underlying factors such as autonomic dysfunction, antihypertensive therapy, inflammation, posture, and stress, could contribute to HT [118].

#### 6.2.2. Hypertension Treatment to Prevent HT

Guidelines recommend maintaining BP < 180/105 mmHg throughout the initial 24 h period following thrombolytic therapy [63]. In AIS patients with uncontrolled blood pressure, initiating treatment with intermittent boluses versus a continuous infusion did not significantly alter the time to alteplase administration [119]. In addition, no significant difference in time to alteplase was observed between labetalol and nicardipine [120].

The ENCHANTED study evaluated whether aggressive early BP reduction (SBP 130–140 mmHg within 60 min) improves outcomes compared with conventional management (SBP < 180 mmHg) in IV tPA–treated ischemic stroke [121]. The intensive BP strategy was associated with a lower 7-day incidence of any ICH compared with guideline-recommended management [121]. Despite evidence that early BP control can affect hemorrhagic risk, an optimal BP goal remains undefined, and no consensus threshold exists [121].

However, a significant importance was attributed to the thresholds of 140 mm Hg and 180 mmHg, with the appearance of a U-shaped pattern between SBP and outcomes in stroke. Poorer prognosis has the patients who are at the two extremes, SBP between 140 and 180 mmHg being considered optimal [122].

Nevertheless, the limits are broad, particularly because an increased BP may favor perfusion through collaterals, especially in the case of occlusion of a large-caliber vessel. Cerebral circulation regulation is particularly vulnerable in the first 48 h, the so-called hyperacute phase, where intensive BP lowering may impair it [122]. In the study of Rasmussen et al., intensive BP lowering was safe, despite a reduction in intracranial hemorrhage; clinical outcomes were not improved relative to those achieved with guideline-level blood pressure control [123]. In other studies, decreased SBP was associated with improved neurological status and lower sICH rates after rt-PA treatment [124,125].

Even though, as previously noted, the thresholds are not rigid, hemodynamic factors remain closely linked to the risk of HT [107]. The consensus, however, is that lowering the post-reperfusion BP value is essential for ensuring the vitality of the penumbra zone [126]. Maintaining cerebral blood flow to the penumbra in acute stroke depends critically on preserved autoregulatory capacity [127]. Impairment of cerebrovascular autoregulation in stroke is associated with a slow recovery trajectory, with restoration often taking up to 3 months [128,129].

In the setting of partial reperfusion, maintaining a higher arterial pressure can help preserve collateral flow to the ischemic bed and mitigate infarct growth in HI-1/HI-2 cases [130]. In case of complete recanalization, the guidelines recommend strict BP control [126]. Stricter BP targets might reduce the likelihood of hematoma expansion among patients with parenchymal hematoma [17].

**Table 2:** Studies Assessing Thrombolysis Blood Pressure Metrics and sICH Risk in AIS.

Study (First Author, Year)	Country/Region	Design	Sample Size (N)	Population (AIS + IVT Only/IVT + EVT)	IVT Agent	Conclusions	Citation (PMID/DOI)
Waltimo 2016	Finland	Observational cohort	1868	AIS + IVT only	Alteplase	Higher systolic BP at multiple post-IVT time points (2, 4, 12, 48 h) was associated with sICH; per +10 mmHg, OR ≈ 1.14 (2–4 h) and 1.12 (12–48 h). No significant difference at 24 h. sICH (ECASS II) overall 5.8%.	[94]
Mokin 2012	USA	Retrospective cohort	267	AIS + IVT only	Alteplase	Among patients who developed symptomatic ICH after IV tPA, higher post-tPA systolic BP correlated with larger initial hematoma volume (r = 0.46; *p* = 0.03). Greater reductions in SBP were associated with less hematoma growth (r = −0.67; *p* = 0.02); diastolic BP showed no similar association.	[95]
Delgado-Mederos 2008	Spain	Prospective cohort	80	AIS with MCA occlusion; IVT only	Alteplase	Higher short-term BP variability (SD of SBP/DBP over 24 h) was linked to greater DWI lesion growth, worse early clinical course, and poorer 3-month outcome; effects were pronounced in patients without early recanalization.	[96]
Butcher 2010 (EPITHET)	International (EPITHET trial)	Post hoc analysis of randomized trial (tPA vs. placebo)	97 (49 tPA; 48 placebo)	AIS randomized to IV tPA vs. placebo (IVT context)	Alteplase	24 h time-weighted mean SBP independently predicted parenchymal hematoma. Baseline SBP did not differ by HT status. PH occurred more often with tPA vs. placebo (11/49 vs. 4/48; *p* = 0.049).	[97]
Sun 2020	China	Prospective observational cohort	306	AIS + IVT only	Alteplase	Independent predictors of HT included age ≥ 68 years, smoking, atrial fibrillation, NIHSS ≥ 17, and systolic BP at two hours ≥ 149 mmHg.	[98]
Lei 2022	China	Retrospective cohort; IVT vs. no IVT in AIS with cerebral microbleeds	220 (CMB-positive AIS)	AIS with CMB; IVT subgroup vs. non-IVT controls	Alteplase	IV rt-PA in CMB-positive AIS improved 7-day NIHSS and 90-day good outcome vs. no IVT, with no significant differences in ICH incidence or mortality. Independent risk factors for HT after thrombolysis included longer onset-to-needle time, higher baseline NIHSS, and atrial fibrillation.	[99]
Ahmed 2009 (SITS-ISTR)	International (SITS-ISTR registry)	Retrospective analysis of a prospective registry	11,080	AIS treated with IV thrombolysis	Alteplase	Higher systolic BP between 2 and 24 h post-thrombolysis was linked to worse outcomes; categorical SBP showed a linear relation with sICH and a U-shaped relation with mortality and independence (best outcomes at SBP 141–150 mmHg). Withholding antihypertensives up to 7 days in patients with prior hypertension was associated with higher sICH and mortality, whereas initiating therapy in newly recognized moderate hypertension was associated with lower mortality and similar sICH compared with no treatment.	[100]
Zhu 2025	China	Prospective cohort	340	AIS + IVT only	Alteplase	Over the first 24 h post-thrombolysis, higher average pressures (SBP/MAP) tracked with worse 90-day outcomes; each +10 mmHg in mean SBP was linked to ~20% higher odds of mRS 3–6. The poorer-outcome group showed greater BP variability Larger SBP reductions in the 0–2 h and 2–6 h windows were associated with less ICH at 24 h, without a corresponding gain in 90-day functional status.	[103]
Reddy 2023	India	Retrospective analysis of a prospective registry	237	AIS + IVT only	Alteplase	A prior stroke, a baseline NIHSS > 15, a mean SBP ≥ 160 mmHg, and SBP variability > 45 were each independently associated with higher odds of 3-month disability (mRS > 2). Symptomatic ICH occurred in 11 patients (4.6%). Factors linked to sICH included age > 60 years, atrial fibrillation, admission glucose ≥ 180 mg/dL, and SBP variability > 45.	[104]
Liu 2024	China	Retrospective cohort	138	AIS + IVT only	Alteplase	HT occurred in 39.1%. The risk of post-thrombolytic HT was associated with excessive 24 h systolic BP extremes after admission, independent of BP at the time of thrombolysis and post-thrombolysis. (OR = 1.042; 95% CI: 1.000–1.086, *p* < 0.05).	[112]
Kamp 2022	USA	Multicenter retrospective cohort	179	AIS + IVT only;	Alteplase	In AIS patients presenting with uncontrolled BP, starting with intermittent boluses versus a continuous infusion did not meaningfully alter the time to alteplase.	[119]
Huang 2025	USA	Multicenter retrospective cohort	481	AIS + IVT only;	Alteplase	No meaningful difference in door-to-needle time was observed between labetalol and nicardipine.	[120]

### 6.3. Hyperglycemia

Type 2 diabetes mellitus, in particular, is a determinant in the development of cardiovascular disease [131]. Hyperglycemia is also present in up to 40% of patients with AIS, this being seen in two major circumstances: either a result of a known pre-existing diabetes mellitus or as “stress hyperglycemia” associated with a rise in cortisol and catecholamines [132,133,134]. Moreover, patients with hyperglycemia and AIS present larger infarct size with more severe ischemia and also a higher risk of HT [135,136,137,138,139,140]. In addition to an increased risk of hemorrhagic transformation, patients with hyperglycemia also experience worse short- and long-term clinical outcomes/mortality and a higher rate of recurrence [21,64,65,141,142,143]. In the Multicenter rt-PA Stroke Survey, diabetes was identified as the only clinically significant factor associated with symptomatic ICH [144]. Glucose level, quantified in 50 mg/dL increments, was a strong predictor of all ICH subtypes [144]. In the SITS study, among nine independent risk factors, baseline glucose level of ≥180 mg/dL was associated with an increased risk of symptomatic intracerebral hemorrhage [145].

However, the 140 mg/dL threshold was recognized in many articles for its clinical significance [139,146]. Patients with admission blood glucose exceeding 140 mg/dL had a worse prognosis and were at higher risk of developing sICH than those with blood glucose on admission < 140 mg/dL [146]. Another study proposes a value of 200 mg/dL for quantifying the risk of disability and death [147]. Both acute and chronic hyperglycemia are predictive of poor outcomes and high mortality in AIS patients treated with tPA [146], consistent with studies in rats with type 1 [138] and type 2 diabetes [136]. Notably, studies have established that treating diabetes with sulfonylurea (SU) drugs provides protection against brain swelling and the occurrence of hemorrhagic transformation. This is expected due to the involvement of the SUR1 receptor (sulfonylurea receptor 1) in microvascular dysfunction [66]. Given their poor prospects and higher risk of bleeding after therapy, diabetic patients are especially likely to benefit from continued research into novel therapeutic strategies.

Although, as previously mentioned, many studies link hyperglycemia to poor prognosis and high mortality in AIS patients treated with tPA, certain studies found no significant association between the presence of diabetes and the rate of hemorrhage. However, these patients still experienced poorer clinical outcomes [148].

Regarding the mechanisms by which hyperglycemia influences the development of hemorrhagic transformation, these are not completely understood. However, a correlation between Type 2 DM and increased blood levels of PAI-1 has been demonstrated on multiple occasions [67]. Further imbalance is noted as hyperglycemia also increases thrombin levels and stimulates the intrinsic pathway [68]. All of these factors suggest, in one aspect, an increase in coagulability and a decrease in the functioning of tPA treatment. Nonetheless, hyperglycemia exacerbates the hypoperfusion and hypoxia of the arterial wall, rendering it more susceptible to degeneration and necrosis [149]. Hyperglycemia might lead to BBB disruption, followed by increased permeability [69]. All these factors, taken together, promote the incidence of HT, primarily through the effects of hyperglycemia on coagulation, vascular wall remodeling, and its interaction with rtPA [150].

### 6.4. Dyslipidemia

Although the link between long-term exposure to elevated levels of low-density lipoprotein cholesterol (LDL-C) and atherosclerotic cardiovascular disease (ASCVD) has been intensively studied with equivocal results, the association of dyslipidemia with HT of AIS has not been clearly elucidated to date [151].

In acute events and certain diseases, a phenomenon known as the lipid paradox has been observed [152]. This suggests that lower lipid levels within the first 24 h correlate with unfavorable outcomes, a phenomenon consistently observed in patients with AIS [70]. On the other hand, in hemorrhagic infarction, the lipid profile does not seem to influence the mortality [70]. Similar results can be observed in the case of HT following reperfusion in ischemic stroke, where low total cholesterol on admission does not seem to correlate with HT [153].

Nonetheless, the findings remain conflicting, as some evidence implies an association between reduced cholesterol levels and a higher risk of symptomatic bleeding [71]. Moreover, while low LDL-C levels were associated with HT, and higher levels of high-density lipoprotein cholesterol (HDL-C) showed a positive correlation with HT, a higher overall LDL-C/HDL-C ratio was correlated with an increased risk of HT [154]. The exact mechanism remains unclear, but cholesterol levels are known to play a key role in maintaining the stability of the microvasculature [155].

The matter is proving to be even more complex regarding statin treatment, as patients treated with statins tend to exhibit lower stroke severity [72]. However, a European multicenter study revealed that this effect is lost in those receiving IVT [156]. Some studies identified statin use as predictive of sICH after IVT [28,157,158,159,160]. Nonetheless, the results were not validated in a study involving over 20,000 patients, nor in corresponding animal models [73,161].

### 6.5. Smoking, Sex, and Body Weight

An important behavioral aspect, namely smoking, has also been studied for its known implications in cardiovascular pathology, including AIS. Persistent smokers after an AIS face a higher risk of cardiovascular events and mortality [74]. Smoking patients with AIS receiving rt-PA have also been identified as having a higher risk for HT. The suggested mechanism was the nicotine effect on the adrenal gland with cholinergic involvement, resulting in increased blood pressure and HT [75]. Smoking is also associated with alterations in the coagulation cascade and vascular dysfunction, leading to atherosclerosis, hypertension, and a cumulative prothrombotic effect [75].

Contrary to smoking, a non-modifiable risk factor could be considered gender. The data is even more inhomogeneous, with some studies claiming that male gender favors HT and, on the other hand, proposing female gender as an unfavorable prognostic factor [61]. The same contradictory results were identified in the case of body mass index, with some studies even describing what is called the “paradoxical effect of obesity” or, rather, in the present context, the “bleeding-obesity paradox” [76]. Under these circumstances, the risk of HT was found to be lower in obese patients [61].

## 7. Conclusions

Metabolic energy failure during ischemia initiates excitotoxic calcium influx, oxidative stress, and inflammatory signaling that converge on endothelial injury and breakdown of the BBB. Hypoxia destabilizes tight junctions and the basement membrane, while impaired cerebrovascular autoregulation magnifies vulnerability to reperfusion. These mechanisms—spanning core and penumbra—provide a biologic rationale for the observed links between early hemodynamic instability, metabolic dysregulation, and the risk of hemorrhagic transformation after thrombolysis.

Among cardiovascular risk factors, age, hypertension, diabetes, TC, LDLC, HDL-C, and TG were statistically significant predictors of ICH after thrombolysis. Lower evidence of association exists between body weight, sex, dyslipidemia, and very low evidence of association between smoking and HbA1c, and the risk of ICH.

In summary, the most actionable predictors of hemorrhagic transformation after IV thrombolysis are elevated post-tPA systolic pressure (2–24 h) and greater short-term BP variability—warranting SBP < 180/105 mmHg with avoidance of large oscillations—together with prompt identification and controlled treatment of admission hyperglycemia (≥140–180 mg/dL), while older age and multimorbidity merit intensified monitoring, and lipid/statin signals remain heterogeneous such that routine statin discontinuation is not justified and decisions should be individualized.

In the years to come, the desirable goal of improving both reperfusion rates and functional outcomes, while reducing hemorrhagic complications, may become achievable rather than remain an illusion, thanks to ongoing research innovations.

## Figures and Tables

**Table 1:** Summary of the main cardiovascular risk factors, their mechanisms influencing HT in AIS, and recommended management strategies.

Risk Factor	Modifiable/ Non-Modifiable	Mechanism	Effect on HT/Recommendations
Age	Non-modifiable	Arteriosclerosis and microangiopathy interfere with coagulation. Comorbidities such as cerebral amyloid angiopathy or hypertensive microangiopathy further compromise vessel stability	Increases the risk of HT, particularly after Alteplase treatment [13,59].
Sex	Non-modifiable	It is believed that gender alone is not the determining factor, but rather the combination of several independent factors; for example, the degree of enrollment, women are less likely to be enrolled in trials for intracerebral hemorrhage.	The results are contradictory, but there may be a higher morbidity in women compared to men [60,61].
Arterial hypertension	Modifiable	Impaired cerebral autoregulation in ischemic tissue, making perfusion pressure-dependent. Exacerbates inflammation, induces vascular remodeling, and alters collateral flow and endothelial function.	BP < 180/105 mmHg throughout the initial 24 h period following thrombolytic therapy [62,63].
Diabetes/Hyperglycemia	Modifiable	Increased coagulability, hypoperfusion, and hypoxia of the arterial wall and increased permeability at the level of the BBB.	Most studies associate admission hyperglycemia, whether or not related to previously diagnosed diabetes, with the occurrence of HT, its severity, and short- or long-term outcomes [64,65,66,67,68,69].
Dyslipidemia	Modifiable	Cholesterol levels are known to play a key role in maintaining the stability of the microvasculature.	The findings remain conflicting, with studies showing that reduced cholesterol levels are associated with a higher risk of symptomatic bleeding and others showing no correlation. This aspect remains equally uncertain in the context of statin therapy [70,71,72,73].
Smoking	Modifiable	Cholinergic involvement, alterations in the coagulation cascade, and vascular dysfunction, leading to atherosclerosis.	Persistent smokers after an AIS face a higher risk of cardiovascular events and mortality [74,75].
Obesity	Modifiable	Obese patients have higher levels of multiple coagulation factors and also exhibit suboptimal responses to antithrombotic therapy, which could limit post-thrombolysis bleeding. Furthermore, obese patients show fewer cases of severe stroke or cardioembolic etiology.	The risk of HT was found to be lower in obese patients [61,76].

## Data Availability

No new data were created or analyzed in this study. Data sharing is not applicable to this article.
